# Atrial Cardiomyopathy: From Diagnosis to Treatment

**DOI:** 10.31083/RCM25124

**Published:** 2025-01-20

**Authors:** Zheyu Liu, Tao Liu, Gang Wu

**Affiliations:** ^1^Department of Cardiology, Renmin Hospital of Wuhan University, 430060 Wuhan, Hubei, China; ^2^Cardiovascular Research Institute, Wuhan University, 430060 Wuhan, Hubei, China; ^3^Hubei Key Laboratory of Cardiology, 430060 Wuhan, Hubei, China

**Keywords:** atrial cardiomyopathy, atrial fibrillation, atrial remodeling, cardiogenic embolism

## Abstract

With a better understanding of the susceptibility to atrial fibrillation (AF) and the thrombogenicity of the left atrium, the concept of atrial cardiomyopathy (ACM) has emerged. The conventional viewpoint holds that AF-associated hemodynamic disturbances and thrombus formation in the left atrial appendage are the primary causes of cardiogenic embolism events. However, substantial evidence suggests that the relationship between cardiogenic embolism and AF is not so absolute, and that ACM may be an important, underestimated contributor to cardiogenic embolism events. Chronic inflammation, oxidative stress response, lipid accumulation, and fibrosis leading to ACM form the foundation for AF. Furthermore, persistent AF can exacerbate structural and electrical remodeling, as well as mechanical dysfunction of the atria, creating a vicious cycle. To date, the relationship between ACM, AF, and cardiogenic embolism remains unclear. Additionally, many clinicians still lack a comprehensive understanding of the concept of ACM. In this review, we first appraise the definition of ACM and subsequently summarize the noninvasive and feasible diagnostic techniques and criteria for clinical practice. These include imaging modalities such as echocardiography and cardiac magnetic resonance imaging, as well as electrocardiograms, serum biomarkers, and existing practical diagnostic criteria. Finally, we discuss management strategies for ACM, encompassing “upstream therapy” targeting risk factors, identifying and providing appropriate anticoagulation for patients at high risk of stroke/systemic embolism events, and controlling heart rhythm along with potential atrial substrate improvements.

## 1. Introduction 

Atrial fibrillation (AF) is one of the most common cardiac arrhythmias in clinical practice, affecting over 50 million individuals worldwide [1, 2, 3]. With the aging population and increasing life expectancy, the incidence and prevalence of AF are rising and are expected to more than double over the next 30 years [2, 4]. Due to its serious health implications and significant economic burden on families and society, AF has become a serious global public health issue [2, 4]. Over the last few decades, considerable progress has been made in understanding the pathogenesis of AF. The mechanism of AF is a complex process involving multiple factors and molecular mechanisms. Current research generally agrees that the occurrence and maintenance of AF are due to the combined effect of triggers [5] and substrates [6, 7]. In recent years, the concept of atrial cardiomyopathy (ACM) has been increasingly recognized, highlighting abnormal structural and functional changes in the atria that may be associated with the occurrence and maintenance of AF. The pathophysiological processes of ACM may include atrial fibrosis, atrial conduction abnormalities and atrial contractile dysfunction [8]. These changes can promote the atrial electrical and structural remodeling, providing the triggers and substrates for the onset and maintenance of AF [9]. Additionally, the persistent presence of AF may further exacerbate ACM, creating a vicious cycle [10]. Despite its importance, ACM still remains a scientific concept that has not yet been integrated into routine clinical practice. Many clinical practitioners still have gaps in their understanding and management of ACM. This review aims to provide clinical practitioners with a deeper understanding of the definition, diagnosis, and management of ACM, and to offer patients more specialized treatment approaches and management strategies.

## 2. Definition of Atrial Cardiomyopathy

In 1972, Williams *et al*. [11] described a familial disease characterized by atrial arrhythmias and atrioventricular block and first used the term “*atrial cardiomyopathy*” to define this condition. Over the past half century, with the deepening understanding of ACM, this concept has evolved from an initial descriptive definition to a well-recognized medical term. According to the EHRA/HRS/APHRS/SOLAECE expert consensus, ACM is defined as “any complex of structural, architectural, contractile or electrophysiological changes affecting the atrium with the potential to produce clinically relevant manifestations” [12]. This definition remains relatively vague and lacks clear diagnostic criteria. It is important to distinguish ACM from “*atrial remodeling*”. Atrial remodeling refers to the adaptive regulation of cardiomyocytes in response to external stressors (e.g., hypertension, heart failure, AF, and others), to maintain homeostasis [13]. These changes, encompassing atrial wall thickening, atrial dilation, and alterations in atrial electrophysiological characteristics, initially serve to compensate for cardiac function, but they may gradually evolve into nonadaptive changes, leading to progressive atrial pump failure and atrial arrhythmias [13]. The concept of ACM emphasizes the pathological changes within the atrial myocardium, which may serve as either the direct cause or the consequence of atrial remodeling. From this perspective, atrial remodeling can be considered a specific phase of ACM, particularly as a manifestation of ACM progression. However, ACM is a broader concept, encompassing not only atrial remodeling but also other inherent pathological changes in the atrial myocardium, including genetically related ACM [14] as well as systemic diseases (e.g., amyloidosis) involving the atria [15].

## 3. Diagnosis of Atrial Cardiomyopathy

A key reason why clinical practitioners have an insufficient understanding of ACM is the lack of clear diagnostic criteria. According to the EHRA/HRS/APHRS/SOLAECE expert consensus, the classification of ACM relies on histopathological findings and the presence of fibrosis and/or non-collagen deposits [12]. This pathological diagnosis method is impractical for clinical application. Here, we briefly introduce commonly used auxiliary inspection methods and feasible diagnostic criteria for assessing ACM. Effective assessment and characterization of ACM progression could enable a more informed and individualized strategy for addressing modifiable risk factors, as well as for the primary prevention of ACM complications.

### 3.1 Imaging Examination for Atrial Cardiomyopathy 

Due to the widespread use of echocardiography, it has become the primary method for assessing ACM. Left atrial (LA) dilation is the most prominent feature in the remodeling process of ACM and the visual representation of LA diastolic dysfunction [16]. It is a significant predictor of various cardiovascular diseases, such as AF, stroke, and heart failure [17]. The measurement of LA anteroposterior diameter through M-mode and subsequent two-dimensional echocardiography is simple and reproducible, previous studies have used LA anteroposterior diameter as one of the morphological parameters for ACM [12, 18]. However, due to the irregular three-dimensional structural characteristics of the atrium and the heterogeneity of atrial remodeling, measuring the atrial volume is more accurate for reflecting and assessing the true state of the atria than atrial diameter. In the case of LA enlargement, the increase in LA anteroposterior diameter is not directly proportional to the increase in the total LA volume, which becomes the main source of error in the assessment of LA volume by two-dimensional echocardiography [19]. LA volumes assessed by two-dimensional echocardiography are generally less than those calculated by cardiac computed tomography (CCT) or by cardiac magnetic resonance imaging (CMRI) [17]. On the contrary, the accuracy of three-dimensional echocardiography in evaluating LA volume is closer to CCT or CMRI [10]. The previous view was that LA maximum volume is the most definitive parameter of LA morphology and reflects LA diastolic function to a certain extent [20]. However, the latest research supports the important role of minimal LA volume in assessing atrial function and disease [21]. Atrial fibrosis can lead to atrial structural remodeling, which is closely related to changes in atrial volume [22]. Another study has shown that LA enlargement is associated with adverse clinical outcomes, independent of AF, such as stroke and heart failure [23, 24]. However, in addition to intrinsic LA pathology, LA volume is also associated with a variety of factors, including left ventricular filling pressure, valvular heart disease, and so on, therefore it is difficult to separate the impact of LA dilatation from left ventricular (diastolic) dysfunction [17]. Moreover, changes in LA volume are reversible, thus LA volume does not necessarily reflect LA function independently [25].

**Fig. 1.**
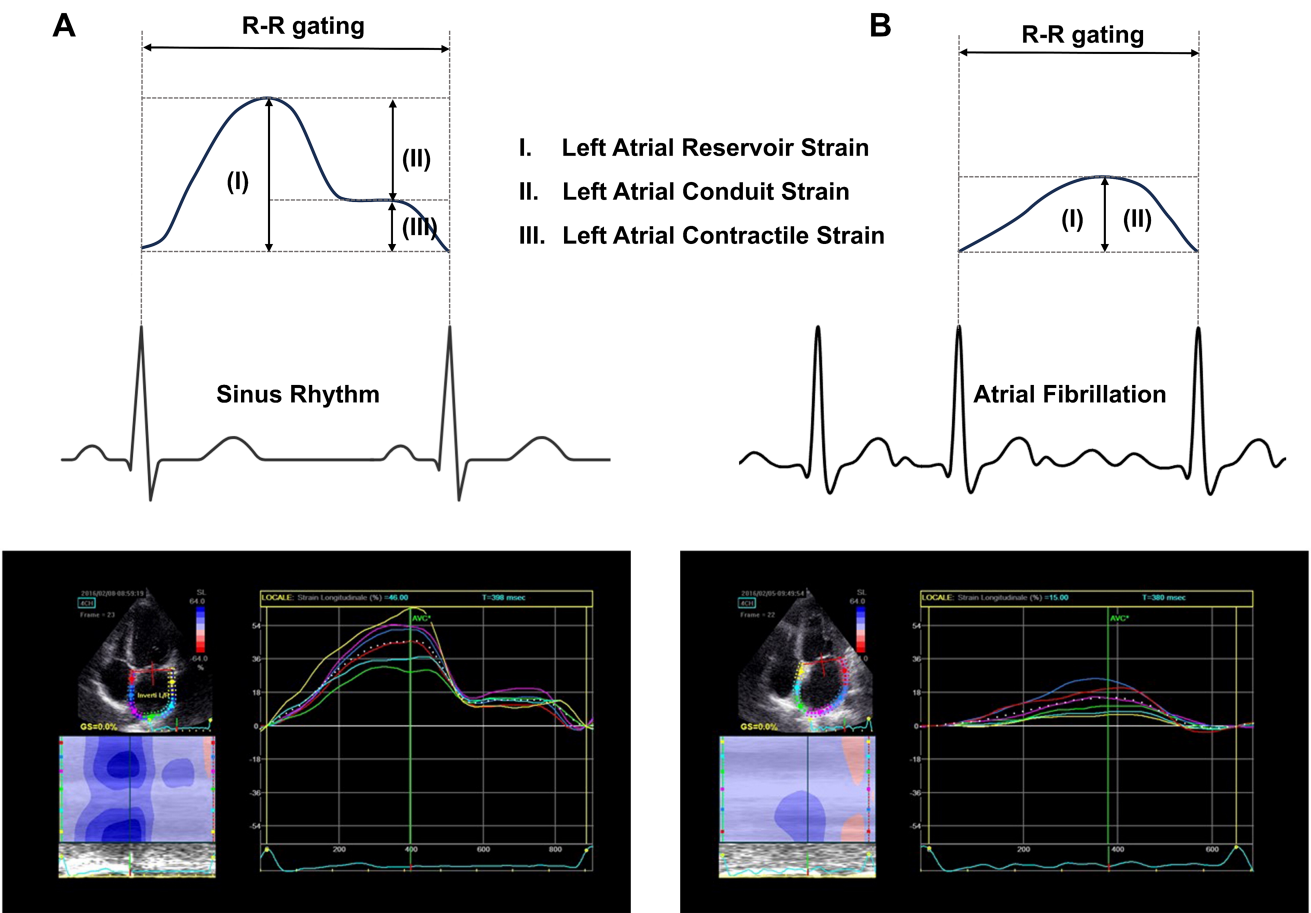
**Schematic of left atrial strain in sinus rhythm (A) and atrial fibrillation (B)**. (A) During sinus rhythm, LAS_r_ = –(LAS_cd_ + LAS_ct_); (B) During atrial fibrillation, LAS_r_ = –LAS_cd_ due to the absence of LAS_ct_. LAS_r_, left atrial reservoir strain; LAS_cd_, left atrial conduit strain; LAS_ct_, left atrial contractile strain. Partially adapted from Cameli *et al*. [35].

In addition to changes in LA structure, LA dysfunction also provides important diagnostic clues for ACM. In two prospective studies, after adjusting for baseline risk factors, a decreased left atrial emptying fraction (LAEF) was identified as a significant risk factor for new-onset AF or atrial flutter, independent of LA volume and left ventricular ejection fraction (LVEF) [26, 27]. Furthermore, a decreased LAEF was also associated with low-voltage zones in electroanatomical mapping [28] and the recurrence of AF after radiofrequency ablation [29]. Eichenlaub *et al*. [30] used a low-voltage zone (<0.5 mV) greater than 2 cm^2^ detected by electroanatomical mapping as the diagnostic criterion for ACM and assessed the value of LAEF in diagnosing ACM. Their results indicated that a LAEF cutoff point of less than 34% could predict ACM effectively (area under the curve [AUC] 0.846, sensitivity 69.2%, specificity 76.5%) and was associated with a higher risk of AF recurrence after radiofrequency ablation [30]. Furthermore, LA flow status can also serve as a predictive indicator for ACM, with low left atrial appendage (LAA) peak flow velocity being associated with the recurrence of AF after ablation [31] and multiple infarctions in patients with cryptogenic stroke [32]. Another echocardiographic parameter that has gained attention is the LA strain parameter. Compared to conventional echocardiographic measurements, LA strain obtained through two-dimensional speckle-tracking echocardiography offers several advantages: it is free from angle alignment issues and is less influenced by loading conditions [33]. Additionally, LA strain can detect functional abnormalities even in the absence of visible LA dilation, making it a valuable predictor for ACM. The LA function consists of three primary components: the reservoir, the conduit, and the active pump. During a normal cardiac cycle, approximately 75% of the stroke volume flows into the left ventricle while the LA volume decreases during the early- and mid-conduit phases. In the pump phase, the LA actively contracts to expel the remaining 25% of the blood [34]. In the case of AF, the absence of effective atrial contraction typically reduces stroke volume by about 25%. The terminology of “LA strain” varies across different studies and appears as peak atrial longitudinal strain or atrial global strain. In this review, as shown in Fig. 1 (Ref. [35]), we use the terms LA reservoir strain (LAS_r_), conduit strain (LAS_cd_), and contractile strain (LAS_ct_) based on a consensus statement [36]. Previous studies have confirmed that LAS_r_ is inversely correlated with LA fibrosis confirmed histologically (R –0.55~–0.82, *p* < 0.001) [37, 38]. Furthermore, LAS_r_ has a predictive value for newly developed AF and stroke in the general population [39]. LAS_r_ was also independently associated with stroke/systemic emboli (S/SE) events in patients with AF (OR 0.74; 95% CI 0.67–0.82; *p <* 0.001), providing an incremental predictive value over the CHA_2_DS_2_-VASc (congestive heart failure, hypertension, age ≥75 years, diabetes mellitus, prior stroke or transient ischemic attack or thromboembolism, vascular disease, age 65 to 74 years, sex category) score [40]. In a retrospective study, transesophageal echocardiography was utilized to determine the presence of LAA thrombosis in patients with non-valvular AF scheduled for electrical cardioversion. Additionally, transthoracic echocardiography and LA strain parameter measurements were routinely performed. The results of the multivariate logistic regression analysis indicated that LVEF, E/e’ (early diastolic velocity of mitral flow/early velocity of diastolic mitral annulus motion) ratio, and LAS_r_ were all independently associated with LAA thrombosis. Among these, LAS_r_ (cutoff value ≤9.1%, AUC 0.95) demonstrated the best diagnostic performance [41]. Another study found LA strain to be a significant predictor of AF recurrence after ablation over a 6-month follow-up period: patients who maintained sinus rhythm showed greater improvement in LAS_r_ than those who relapsed [42]. However, similar to three-dimensional cardiac ultrasound, LA strain is not widely used yet in current medical facilities, which limits its value in screening and evaluating ACM.

CMRI is precise and highly reproducible and has now become the gold standard for evaluating LA volume and function. Late gadolinium enhancement (LGE) imaging is a non-invasive, well-established technique for detecting atrial fibrosis. The imaging agent gadolinium is taken up and released into the bloodstream quickly by healthy myocardial tissue, but it dissipates more slowly from diseased tissue with interstitial fibrosis. This property allows visualization of the amount of contrast agent retained in the myocardium at a delayed time (10–30 minutes) after contrast injection. Therefore, parametric T1-mapping indices offer high diagnostic accuracy for identifying diffuse interstitial fibrosis [43]. LA remodeling characterized by LGE-CMRI shows a significant correlation with atrial volume, AF type, and recurrence risk [44, 45]. Additionally, LGE-CMRI also contributes for predicting stroke risk in AF patients, LA fibrosis detected through LGE-CMRI was an independent predictor of stroke events and significantly increased the predictive power of the CHA_2_DS_2_-VASc score [46]. McGann *et al*. [47] attempted to identify LA remodeling and stratify individuals who would benefit from AF catheter ablation through LGE-CMRI. They found that extensive LGE (≥30% LA wall enhancement) predicted a poor response to catheter ablation therapy for AF [47]. In recent years, the application of CMRI in catheter ablation procedures for AF has received significant attention. During a two-year follow-up, Quinto *et al*. [48] found that identifying anatomical veno-atrial gaps using LGE-CMRI in repeat catheter ablation procedures could shorten ablation duration (161 ± 52 minutes vs. 195 ± 72 minutes) and decreased the incidence of recurrent atrial tachycardia (30% vs. 61%). Akoum *et al*. [49] utilized LGE-CMRI to assess the baseline level of atrial fibrosis in patients with AF and the level of residual fibrosis outside the scar tissue three months post-ablation. Their findings indicated that the level of residual fibrosis could serve as a predictor for AF recurrence [49]. However, results from the DECAAF II trial, published in the Journal of the American Medical Association (JAMA), found no statistically significant difference in atrial arrhythmia recurrence rates between CMRI-guided fibrosis ablation plus pulmonary vein isolation (PVI) and PVI catheter ablation alone in patients with persistent AF [50]. One potential explanation is that the ablation strategy for fibrotic atria has not been standardized, lacking a definitive ablation endpoint, making it susceptible to subjective influence from the operator. Moreover, several factors further limit the applicability of LGE-CMRI. For instance, the atrial wall is relatively thin compared to the resolution of CMRI, making its imaging signal susceptible to interference from adjacent tissues or “volume effect”. Additionally, the post-processing of acquired data remains non-standardized. Current algorithms for quantifying atrial fibrosis typically rely on signal intensity comparisons with the patient’s own normal atrial tissue or atrial blood pool signal-to-noise ratio, which lack uniform comparability among different individuals. Furthermore, the imaging quantification algorithms currently in use have not yet been correlated with low-voltage zones identified by electroanatomical mapping or with histological fibrosis detection [51].

### 3.2 Electrocardiogram Examination for Atrial Cardiomyopathy 

Electrocardiogram (ECG) examination also played a routine and crucial role in assessing ACM. The P-wave is the summation of the depolarization of the right atria (first third of the P-wave) and the LA (last third of the P-wave). Several P-wave indices (PWIs) in ECG are currently thought to be associated with ACM, such as P-wave dispersion (PWd), the P-wave terminal force in lead V1 (PTFV1), P-wave axis (PWA), P-wave duration (PWD) and the burden of premature atrial contractions (PACs) [52].

PWd refers to the variation between the maximum and minimum P-wave durations measured simultaneously across the 12 leads of a standard ECG [53]. The durations in different leads of the ECG reflect regional heterogeneity in atrial depolarization. Inhomogeneous and discontinuous atrial conductions contribute to increased PWd based on the anisotropic distribution of connections between atrial myocardial fibers [54]. Collagen deposition in the LA myocardium results in decreased LA compliance, which is manifested as prolongation of the PWd on ECG [55]. Additionally, increased PWd is also considered to be connected with compromised mechanical function and enlargement of the LA [56]. Therefore, increased PWd suggests the formation of an atrial substrate conducive to AF and multiple studies have demonstrated a strong correlation between increased PWd and AF in cases of cryptogenic stroke [57, 58].

PTFV1 is defined as the product of the amplitude and duration of the terminal negative deflection of the P-wave in lead V1. Increased PTFV1 has been demonstrated to be correlated with LA abnormalities, such as enlargement, dysfunction and delayed interatrial conduction [59, 60]. Tiffany Win *et al*. [60] conducted a more detailed analysis of the two components of PTFV1 (amplitude and duration of the terminal negative deflection of P-wave), revealing their distinct predictive value. The duration correlates with the degree of atrial fibrosis, whereas the amplitude is tied to the LA mechanical functions such as volume and LA strain [60].

The PWA is defined as the net direction of electrical forces in the atrium, and its normal value is typically considered to be in the range of 0° and 75°. Previous studies have indicated that abnormal PWA is an important predictor of AF [61] and is associated with an increased risk of ischemic stroke [62] and all-cause mortality [63]. Maheshwari *et al*. [64] also found that abnormal PWA was the only PWI linked to an elevated risk of S/SE (HR [hazard ratio] 1.84; 95% CI 1.33–2.55) independent of CHA_2_DS_2_-VASc scores based on two large prospective cohort studies (Atherosclerosis Risk in Communities [ARIC] and Multi-Ethnic Study of Atherosclerosis [MESA]). They also created the P_2_-CHA_2_DS_2_-VASc score, which is a better prediction tool for AF-related ischemic stroke compared to the CHA_2_DS_2_-VASc score [64]. However, a long-term study showed that among subjects with abnormal PWA at baseline, less than 40% continued to have abnormal PWA at follow-up 11 years later [65], suggesting that the measurement of the PWA is quite unstable, limiting its clinical application. Furthermore, only the frontal PWA is routinely reported on a 12-lead ECG, atrial depolarization occurs in three-dimensional space, therefore a three-dimensional PWA loop can more accurately simulate the actual directionality of the atrial depolarization vector *in vivo* [66]. Spatial P loop vectors and morphology and the association between three-dimensional depolarization vector changes and ACM still need further research and demonstration.

In addition to the above PWIs, the prolonged PWD and frequent PACs are also considered to be related to ACM. The prolonged PWD (>120 ms) indicates the presence of interatrial conduction block, which is caused by an altered cardiac conduction due to fibro-fatty transformation of the atrium PWD [59, 67]. Several previous studies have demonstrated that prolonged PWD increases the risk of AF [68] and ischemic stroke [69]. Amplified P-wave duration (aPWD) (40 to 50 mm/mV amplification and 100 to 200 mm/s sweep speed) can provide more valuable information. Jadidi *et al*. [70] found that the aPWD of LA (from -dV/dt to the end of the P-wave in lead V1) is closely related to the LA activation time and low-voltage substrate. Similarly, frequent PACs detected on ECG are also associated with AF development [71] and ischemic stroke [72]. The prolonged P-R interval [73, 74] and P-wave area (PWa) [75], which is the total geometric area under the P-wave in the 12-lead ECG, have also been considered related to ACM. However, their predictive performance and clinical application value still require further evaluation.

Artificial intelligence (AI)-based ECG analysis has become a promising direction for studying and diagnosing ACM. Verbrugge *et al*. [76] used AI-based ECG analysis to identify the presence of underlying ACM in patients with heart failure with preserved ejection fraction (HFpEF), and the results were further evidenced by structural, functional, hemodynamic abnormalities, and long-term risk for incident AF. Similarly, another study showed AI-based ECG analysis can effectively quantify the risk of AF and this predictive capability is complementary to traditional clinical risk factor models [77]. In a retrospective study published in The Lancet, Attia *et al*. [78] trained a convolutional neural network on ECG features of sinus rhythm in patients with a history of AF or atrial flutter, achieving good validation in the test group. However, whether these findings can be generalized to screening high-risk populations for AF still requires large-scale prospective studies.

### 3.3 Serum Biomarkers for Atrial Cardiomyopathy

Although not specific, various serum biomarkers characterizing myocyte injury, inflammation, and fibrosis have been linked to the occurrence and outcomes in ACM and can be used as an auxiliary means to evaluate and diagnose ACM.

N-terminal pro-B-type natriuretic peptide (NT-proBNP) and B-type natriuretic peptide (BNP) are well-known indicators of congestive heart failure due to volume overload and myocardial damage. However, NT-proBNP also shows a strong correlation with echocardiographic parameters of LA remodeling and dysfunction [79]. Patel *et al*. [80] found that higher levels of NT-proBNP and troponin I were independently associated with lower LAS_r_ and LAS_ct_ (*p* < 0.05). Furthermore, increased NT-proBNP may serve as a predictor of LA fibrosis and has been utilized as one of the diagnostic criteria for ACM in some investigations [81, 82, 83]. Elevated BNP and NT-proBNP levels have been linked to an increased risk of cardioembolic stroke independently of AF [84], the biomarker-based ABC (age, biomarkers [high-sensitivity (hs-) troponin and NT-proBNP], and clinical history of prior stroke/transient ischemic attack) stroke score was well applicable and generally more suitable than the CHA_2_DS_2_-VASc and ATRIA (Anticoagulation and Risk Factors in Atrial Fibrillation) stroke scores in anticoagulated patients with AF [85]. Furthermore, a meta-analysis by Xu and Tang [86] demonstrated that baseline BNP/NT-proBNP levels can serve as predictive indicators for AF recurrence following successful electrical cardioversion.

In contrast to NT-proBNP and BNP, which are generated by both the atria and ventricles, atrial natriuretic peptide (ANP) is predominantly produced by the atria, theoretically making it a more precise biomarker for assessing ACM. Midregional pro-A-type natriuretic peptide (MR-proANP) is more stable than ANP, leading to its widespread use in clinical settings. Similar to NT-proBNP, MR-proANP levels are independently linked to LA enlargement [87]. A study involving 190 patients demonstrated that MR-proANP is a stronger biomarker than NT-proBNP for indicating the LA volume index and atrial volume overload in individuals with HFpEF [88]. Seewöster and colleagues [89] developed an innovative biomarker-based score (consisting of age 65 or above, NT-proANP levels above the 75th percentile in peripheral blood [≥17 ng/mL], and persistent AF), which was found to be a noticeable predictor of low-voltage zones in patients with AF who were undergoing catheter ablation.

Biomarkers of fibrosis and inflammation are also implicated in atrial inflammation, matrix remodeling, and atrial fibrosis, significantly associated with the progression of ACM. In a sub-analysis of ARTEMIS trail, the soluble isoform of suppression of tumorigenicity-2 (sST2) and hs-C-reactive protein (CRP), retained significant power in predicting new-onset AF after correcting for other risk factors [90]. Additionally, ST-2 is also associated with increased LA volume index [91]. A study including 63 patients showed a negative correlation between galectin-3 and LA related echocardiographic parameters, including LA volume and LA strain rate [92]. Serum levels of carboxyl terminal peptide from pro-collagen I (PICP) and amino terminal peptide from pro-collagen III (PIIINP) have been reported to be associated with the existence of LA fibrosis and may serve as predictors for post-operative AF even in patients without previous AF history [93]. Furthermore, there is also a linear correlation between serum PICP levels and the extent of LA fibrosis [93]. However, the translational value of biomarkers of fibrosis and inflammation in assessing and diagnosing ACM needs further validation.

### 3.4 Electrophysiological Examination for Atrial Cardiomyopathy

Electroanatomical mapping has become a common method for assessing the atrial substrate during AF radiofrequency ablation. This technique involves mapping thousands of bipolar voltage points (i.e., the voltage difference between two adjacent unipolar electrodes) onto a three-dimensional model of the atrium. Current views suggest that low bipolar atrial voltage may serve as an alternative marker for atrial fibrosis. Physiologically, low-voltage zones in electroanatomical mapping result from the anisotropic distribution of connections between cardiomyocytes and fibroblasts, as well as the low coupling between different cell types [94, 95]. Therefore, decreased conduction velocity may also coexist with low-voltage zones. Compared to paroxysmal AF, patients with persistent AF have a longer atrial activation time, which is negatively correlated with LA voltage [96]. Additionally, a reduction in atrial voltage often accompanies increased atrial volume and dysfunction, indicating that low voltage is closely related to changes in LA morphology and atrial wall stress. In an *ex vivo* experiment using Langendorff-perfused canine hearts, acute LA dilation led to a significant decrease in LA voltage, especially in the posterior wall [97]. Another clinical study found that patients with low-voltage zones in the LA typically had larger LA volumes and a higher incidence of persistent AF [98]. Some perspectives suggest that low-voltage zones are also associated with AF triggers. For instance, patients with AF originating from the pulmonary veins often have lower voltages in the pulmonary vein antra, while those with AF triggered from the superior vena cava tend to have lower mapping voltages in the right atrium [99]. Therefore, substrate modification guided by low-voltage zones may be a promising strategy for AF ablation. Rolf *et al*. [100] first reported on this in 2014. In their non-randomized study, AF patients with low-voltage zones who underwent PVI combined with tailored substrate modification achieved a higher 12-month AF-free survival rate compared to those who received PVI alone [100]. Although subsequent studies have yielded conflicting conclusions on whether modification of low-voltage zones benefits patients [101, 102], a meta-analysis concluded that ablation of low-voltage zones can generally improve the long-term effectiveness of AF ablation [103].

**Fig. 2.**
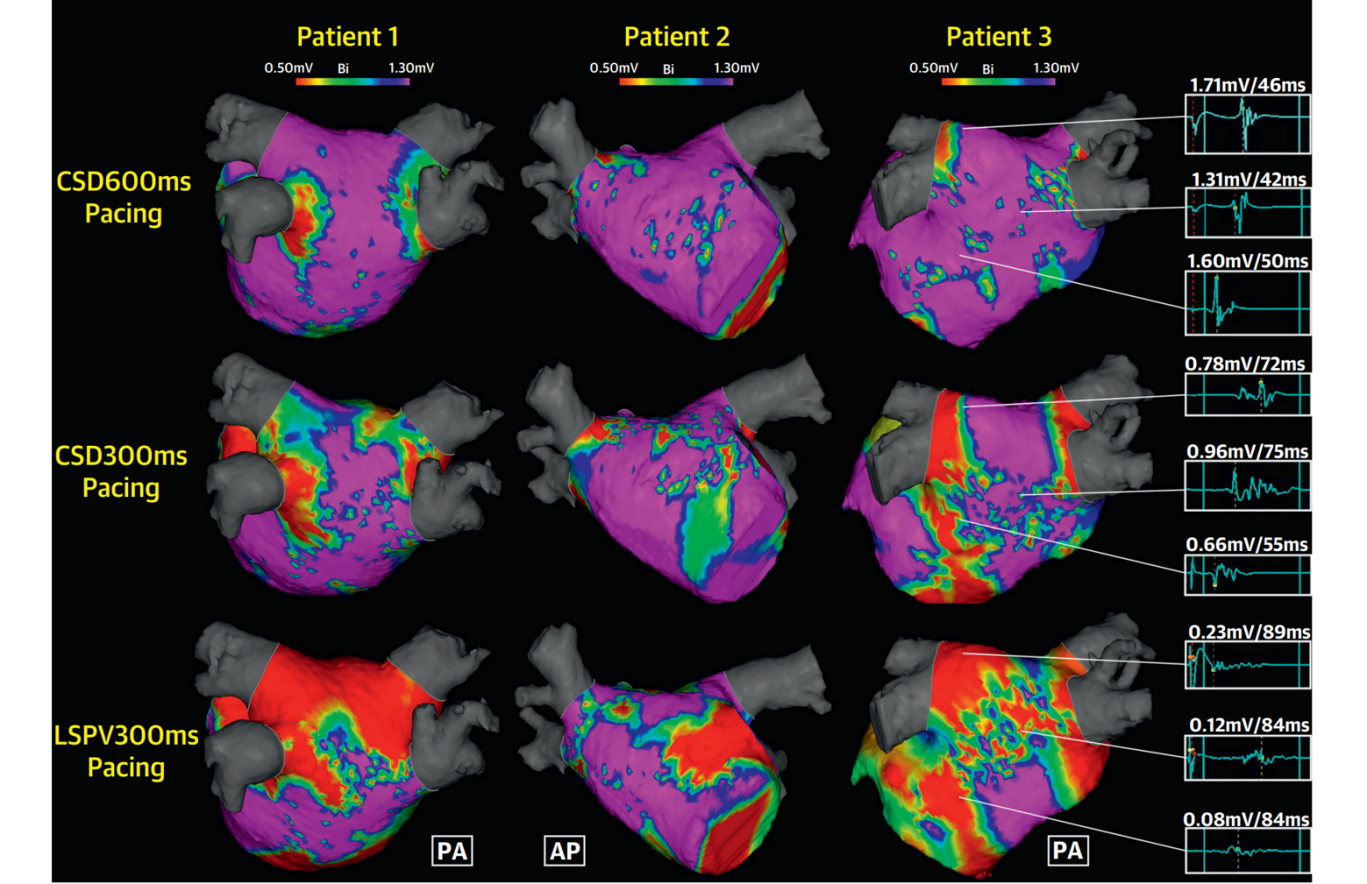
**Marked variation in the mapped atrial substrate was observed under different pacing sites and pacing cycle lengths among three patients**. The red areas represent low-voltage zones (<0.5 mV), which show significant differences with changes in pacing site and cycle length, even within the same patient. In patient 3, a 72-year-old male with persistent atrial fibrillation, the complex fractionated electrograms were longer and accompanied by lower atrial voltages under each identical pacing strategy compared to the other two patients. CSD distal coronary sinus; LSPV, left superior pulmonary vein; PA, posteroanterior; AP, anteroposterior. Adapted from Wong *et al*. [104].

However, electrophysiological examination also has certain limitations in diagnosing ACM. Firstly, as an invasive procedure, it is limited in its widespread application in clinical settings, especially as it cannot serve as a routine screening method for ACM. Secondly, electroanatomical mapping technology has inherent shortcomings in accurately reflecting atrial fibrosis and electrical remodeling. For instance, atrial tissue thickness varies among different patients and different regions of the atrium (such as the pectinate muscles compared to the smooth atrial wall), leading to significant differences in normal voltage levels. Therefore, it is not feasible to simply use a uniform cutoff point (e.g., 0.5 mV) to define low-voltage zones. Furthermore, the distribution of low-voltage zones in electroanatomical mapping is significantly influenced by the direction and velocity of wavefront propagation. Research by Wong *et al*. [104] published in the Journal of the American College of Cardiology (JACC): Clinical Electrophysiology indicates that changes in pacing site and pacing cycle length can significantly affect the low-voltage zones, conduction velocity, and complex fractionation in LA electroanatomical mapping (Fig. 2, Ref. [104]). Additionally, heart rhythm status significantly impacts the presentation of bipolar electrograms. Two early studies revealed that some low-voltage zones detected by electroanatomical mapping during AF episodes unexpectedly displayed normal voltage after the restoration of sinus rhythm [105, 106]. A study by Qureshi *et al*. [107] published in Heart Rhythm, found that with sufficient sampling time, the spatial distribution of low-voltage zones detected during AF episodes correlated more closely with myocardial fibrosis zones identified by LGE-CMRI than those detected during sinus rhythm. The lower voltage observed in bipolar electrograms during AF may be attributed to changes in wavefront direction relative to electrode orientation, as well as the effects of complex fractionation and collision. Compared to bipolar techniques, omnipolar electrograms can extract the maximum atrial voltage during AF episodes without being influenced by these factors [108]. Therefore, the next step should focus on standardizing the pacing site, pacing cycle length, and atrial rhythm as a priority. Based on this standardization, high-quality clinical studies should be conducted to further characterize the auxiliary value of electroanatomical mapping in the diagnosis of ACM and the process of AF ablation. Additionally, we should actively explore and implement new technologies, including omnipolar mapping and dynamic voltage mapping.

### 3.5 Feasible Diagnostic Criteria for Atrial Cardiomyopathy

The inclusion criteria of the ARCADIA trial defined ACM as P-wave terminal force greater than 5000 µV⋅ms in ECG lead V1, a serum NT-proBNP level greater than 250 pg/mL, or LA diameter index of 3 cm/m^2^ or greater on the echocardiogram [83]. The ATTICUS trial compared the effectiveness of apixaban versus aspirin in preventing the recurrence of ischemic stroke. Patients were included based on ECG and echocardiogram parameters indicative of ACM, which included at least one of the following criteria: LA diameter >45 mm (measured at parasternal axis), spontaneous echo contrast in the LAA, LAA flow velocity ≤0.2 m/s, atrial high-rate episodes and CHA_2_DS_2_-VASc score ≥4 [109].

**Fig. 3.**
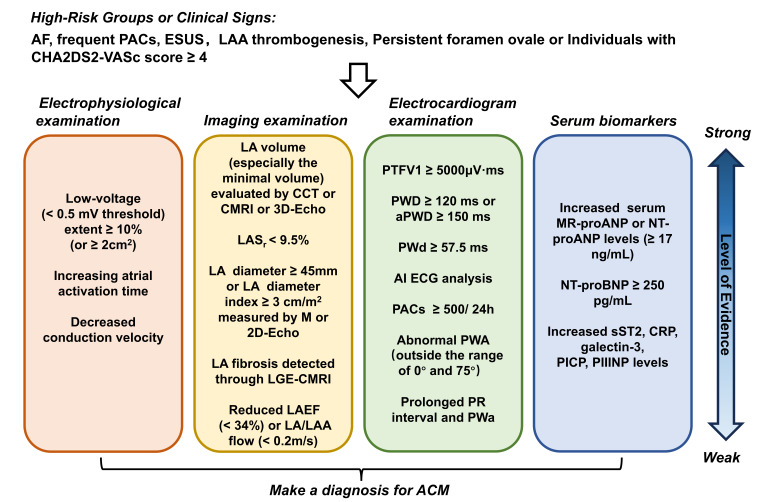
**Diagnostic criteria and evidence levels for atrial cardiomyopathy**. For high-risk individuals or patients with clinical signs of atrial cardiomyopathy, a comprehensive evaluation should be conducted based on the specific circumstances. This evaluation should include imaging examinations, electrocardiograms, serum biomarker assessments, and electrophysiological examinations to accurately diagnose atrial cardiomyopathy. AF, atrial fibrillation; ESUS, embolic stroke of undetermined source; LA, left atrial; LAA, left atrial appendage; CCT, cardiac computed tomography; CMRI, cardiac magnetic resonance imaging; 3D-Echo, three-dimensional echocardiography; LAS_r_, left atrial reservoir strain; LGE, late gadolinium enhancement; LAEF, left atrial emptying fraction; PTFV1, P-wave terminal force in lead V1; PWD, P-wave duration; aPWD, amplified P-wave duration; PWd, P-wave dispersion; AI, artificial intelligence; ECG, electrocardiogram; PACs, premature atrial contractions; PWA, P-wave axis; PWa, P-wave area; NT-proBNP, N-terminal pro-B-type natriuretic peptide; MR-proANP, midregional pro-A-type natriuretic peptide; CRP, C-reactive protein; PICP, carboxyl terminal peptide from pro-collagen I; PIIINP, amino terminal peptide from pro-collagen III; sST2, soluble suppression of tumorigenicity-2; ACM, atrial cardiomyopathy; M, M-mode echocardiography.

Johnson *et al*. [110] established another possible staging system for ACM in the absence of ongoing AF based on a large-scale population survey. Briefly, Johnson *et al*. classified the risk of ACM in individuals into four stages based on ECG and echocardiographic features: Stage 0: No evidence of atrial disease or arrhythmia; Stage 1: PACs >500/24 h OR LA volume index >34 mL/m^2^ OR P-wave abnormalities (P-wave duration >120 ms or terminal P-wave force in V_1_>4 mV⋅s [We believe there is an error here, it should be changed to “mV⋅ms”]); Stage 2: Coexistence of 2 Stage 1 criteria; Stage 3: Coexistence of 3 Stage 1 criteria. Given the relative ease of obtaining ECG and echocardiographic indices, the stratification system proposed by Johnson *et al*. is not difficult to implement in clinical practice. These indices are derived from routine examinations for diagnosing and assessing heart diseases, and their ubiquity and non-invasive nature enable doctors to conduct risk assessments with ease. In the sample, 42% of individuals had at least one marker of ACM, but only 9% had two markers, and 0.3% had three markers. Regrettably, due to the lack of long-term follow-up studies, we are currently unable to accurately determine the risk of AF or other cardiovascular events in individuals at different stages. Based on Framingham Heart Study data, the lifetime risk of AF in the population is 37% [3], indicating that individuals in stage 2 or 3 should be given particular attention. The authors noted that the correlations among the ACM criteria analyzed were relatively weak, with limited overlap [110], indicating that the system required further refinement to enhance the accuracy and reliability of assessment to provide a more systematic and structured framework for the risk evaluation and management of ACM. Based on the above research, we made a summary of the diagnostic criteria and evidence for ACM, as shown in Fig. 3.

## 4. Management Strategies of Atrial Cardiomyopathy

**Fig. 4.**
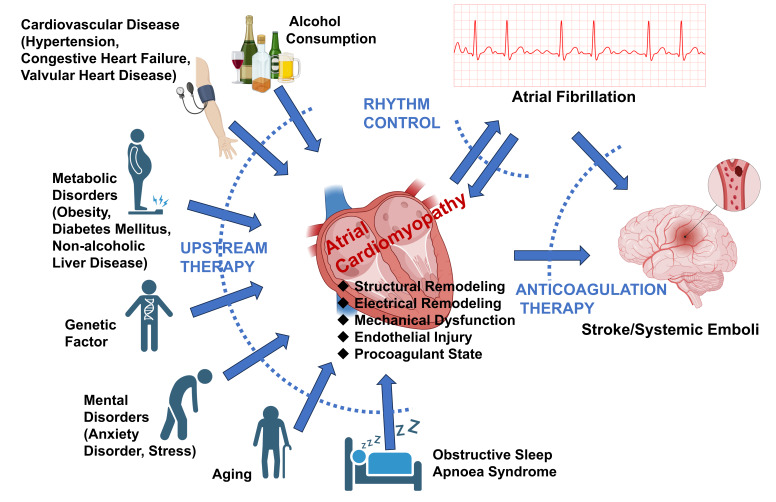
**Major risk factors, complications, and treatment strategies for atrial cardiomyopathy**. Under the influence of various risk factors, the heart may develop into atrial cardiomyopathy characterized by structural and electrical remodeling, mechanical dysfunction, endothelial injury, and a procoagulant state. The main complications of atrial cardiomyopathy include atrial fibrillation and stroke/systemic emboli. Moreover, persistent atrial fibrillation can further exacerbate the atrial substrate for cardiomyopathy. Therefore, the primary treatment strategies for atrial cardiomyopathy include “upstream therapy” to correct risk factors, rhythm control, and prevention of embolic events.

Although there are no unified guidelines or expert consensus specifically tailored to ACM, its management can be informed by relevant clinical practice and research. As shown in Fig. 4, the management of ACM typically includes the following aspects:

### 4.1 Upstream Treatments for Atrial Cardiomyopathy

There are many potential causes or risk factors of ACM, including genetic factors, aging, congestive heart failure, hypertension, valvular heart disease, obstructive sleep apnoea syndrome, alcohol consumption, metabolic disorders (e.g., obesity, diabetes mellitus [DM], and non-alcoholic liver disease), mental disorders (e.g., anxiety disorder, stress) [111]. “Upstream therapies”, including lifestyle changes and risk factor management, should be the cornerstone of ACM management [112]. Shen *et al*. [113] proposed a potential staging system for ACM based on the 2013 American College of Cardiology Foundation/American Heart Association guidelines for heart failure guidelines: Stage A (at risk of developing ACM), Stage B (asymptomatic but detectable ACM), Stage C (manifest disease but potentially reversible), and Stage D (irreversible). Without effective intervention, ACM will eventually progress to stage D. Identifying risk factors in the early stages ACM is crucial, as it allows for the timely initiation of upstream therapies to prevent or even reverse structural and electrophysiological changes in the atrium. Thus, early identification and intervention of reversible risk factors are essential for the primary prevention of ACM complications and represent one of the most important strategies in managing the condition.

Obesity is now widely recognized as being associated with the progression of ACM. The risk of new-onset AF increases by up to 8% with each unit increment in body mass index (BMI) [114, 115]. Although obesity is characterized by the expansion of subcutaneous fat, it is also accompanied by the accumulation of visceral adipose tissue. Epicardial adipose tissue (EAT), a unique visceral fat deposit located adjacent to the heart’s epicardial surface, is now considered a significant factor in the formation and progression of ACM [116]. Venteclef *et al*.’s experimental results [117] indicate that EAT promotes myocardial tissue fibrosis by secreting adipo-fibrokines, such as Activin A. The expansion of EAT also triggers a chronic inflammatory response, with activated adipokines, pro-inflammatory and pro-oxidant cytokines, along with metabolic changes and myocardial alterations, all contributing to the development of ACM [117, 118, 119, 120]. Furthermore, the accumulation of adipocytes within the myocardium can disrupt atrial conduction and facilitate the development and persistence of reentrant circuits [121]. Specifically, these models exhibited LA enlargement, biatrial conduction abnormalities, increased expression of profibrotic factors, exacerbated atrial interstitial fibrosis, and an increased propensity of spontaneous and inducible AF [122]. Existing evidence shows that weight loss can significantly improve symptoms and quality of life in patients with AF, and slow the progression of ACM by reducing EAT [123, 124]. It may also reduce the recurrence of AF after ablation and even reverse persistent AF to paroxysmal AF or normal sinus rhythm [125].

DM is another risk factor for ACM that requires special attention. DM promotes atrial interstitial fibrosis through inflammatory and oxidative stress responses, and it is also accompanied by electrical remodeling, including changes in ion channels and gap junctions [126, 127, 128]. Chao *et al*. [129] conducted a detailed analysis of electrophysiological characteristics using a three-dimensional electroanatomical mapping system in patients with paroxysmal AF who underwent their first catheter ablation. The results showed a significant reduction in atrial voltage in both the right and left atria of patients with diabetes and impaired glucose metabolism, indicating electrical remodeling and atrial fibrosis in individuals with DM. Moreover, the long-term follow-up revealed a significantly higher AF recurrence rate in patients with DM compared to the control group [129]. In addition to structural and electrical remodeling, DM also promotes the progression of ACM by inducing autonomic remodeling [127, 128]. Sprouting and hyperinnervation of the atrial autonomic nerves regulate AF initiation and maintenance through modulation of cardiac electricity working synergistically with electrical remodeling. Furthermore, DM may also contribute to thrombosis in ACM and a higher risk of stroke. Higher levels of Plasminogen activator inhibitor-1 and lower expression of anticoagulant molecules, such as thrombomodulin and protein C, have been described in DM patients [130, 131]. Additionally, DM increases the expression of fibrinogen and tissue factor, enhances thrombin sensitivity and early platelet activation, and induces endothelial damage [128, 132, 133]. Furthermore, effective insulin therapy may alleviate atrial fibrosis and the progression of atrial reentrant substrate [126]. As a novel class of hypoglycemic drugs, sodium-glucose cotransporter 2 inhibitors (SGLT2i) decrease the concentration of plasma glucose by inhibiting glucose reabsorption through the SGLT2 receptor in the proximal tubules of the kidney. Since the 2022 ACC/AHA/HFSA (American College of Cardiology/American Heart Association/Heart Failure Society of America)) Guideline for the Management of Heart Failure recommended SGLT2i for the treatment of chronic heart failure with reduced ejection fraction [134], current evidence also suggests their potential therapeutic value for ACM. In a sub-analysis of the DECLARE-TIMI 58 trial, compared to placebo, dapagliflozin reduced the risk of first AF/atrial flutter or the number of total AF/atrial flutter events by 19% and 23%, respectively, in patients with type 2 DM and cardiovascular injury [135]. A meta-analysis indicated that various SGLT2i could reduce the incidence of AF or atrial flutter, with dapagliflozin providing the greatest benefit [136]. Notably, the protective effects of SGLT2i on ACM are not solely dependent on glucose control. They may also mitigate atrial electrical and structural remodeling by improving mitochondrial function, exerting anti-inflammatory and antioxidant effects [137, 138, 139], and reducing left atrial dilatation through blood pressure reduction and increased natriuresis [138].

In addition, previous studies have shown that alcohol consumption contributes to myocardial fibrosis, left ventricle (LV) enlargement, and the development of AF [140, 141]. Latest research results also indicated that increased alcohol consumption was associated with LA dysfunction, including larger LA volume and lower LA reservoir and contractile strain [142, 143]. Despite the study results indicating a dose-dependent relationship between alcohol consumption and the deterioration of LA volume index and LA strain, it is disappointing to note that the study found no improvement in LA volume index and LV strain after reducing alcohol intake [142], suggesting that atrial remodeling caused by alcohol may be irreversible. However, this does not necessarily mean that limiting alcohol intake is not important for patients with ACM. Current evidence and clinical experience support that reducing alcohol intake can significantly alleviate the AF burden [140].

### 4.2 Predicting and Preventing Stroke/Systemic Embolism Events

The traditional view posits that during AF, the loss of effective contractile function of LA leads to sluggish blood flow, potentially resulting in LAA thrombogenesis and subsequent S/SE, particularly upon the return of atrial contraction with sinus rhythm restoration. However, this traditional perspective has been challenged in recent years. In the ASSERT [144] and TRENDS [145] studies, researchers included 2580 and 2486 patients with implanted monitoring devices, respectively. Among the patients who experienced a S/SE during the follow-up period, only 8% and 23% detected episodes of AF within the 30 days preceding the stroke. Furthermore, the IMPACT trial indicated that promptly initiating anticoagulation therapy upon the detection of atrial arrhythmias (mainly AF) through remote rhythm monitoring by implantable devices did not confer a greater reduction in stroke risk compared to conventional office-based follow-up methods (1.0 vs. 1.6 per 100 patient-years, RR –35.3% [95% CI –35.3%], *p* = 0.251). Moreover, there was no temporal relationship between atrial arrhythmias and S/SE [146]. The outcomes of the aforementioned clinical trials suggest that the relationship between AF and S/SE is not absolute.

The CHA_2_DS_2_-VASc score is currently widely used for S/SE risk identification and stratification in patients with AF [147]. The factors included in this score are precisely the risk factors or primary causes for ACM that are currently widely accepted [12, 148]. However, the intrinsic characteristics of AF (e.g., whether it is persistent, paroxysmal, or long-standing persistent; the frequency of AF episodes if it is paroxysmal; and the characteristics of the f-waves or ventricular rate during AF episodes) do not influence the decision to initiate anticoagulation therapy. In patients without risk factors indicative of ACM, the risk of S/SE in AF patients is similar to that in patients without AF [149]. Therefore, even in patients with persistent AF, anticoagulation therapy is not recommended if the CHA_2_DS_2_-VASc score is 0 [147]. From a different perspective, in patients with amyloidosis and cardiac involvement, the incidence of thromboembolic events can reach as high as 5–10% [15]. Even in sinus rhythm, these patients have a significantly increased risk of developing LA/LAA thrombus [150]. In fact, irrespective of the presence of AF, the inflammatory and fibrotic processes in the LA are the primary causes of impaired atrial conduit function and promote the thrombogenicity of the atrial endocardium [151]. Thus, various lines of evidence suggest that the presence of ACM may be an independent risk factor for S/SE, and early identification of ACM can help predict and prevent the occurrence of S/SE.

Embolic stroke of undetermined source (ESUS) is defined as a non-lacunar cerebral infarction without any large arterial stenoses ≥50% or identifiable cardioembolic causes and accounts for 15%–20% of all ischemic strokes [152]. Traditionally, ESUS was thought to be primarily associated with asymptomatic paroxysmal AF. However, recent research evidence indicates that ACM may be an underlying factor contributing to ESUS. A study comparing the prevalence of ACM among patients with different etiologies of ischemic stroke revealed that the incidence of ACM (defined by PTFV1 ≥5000 µV⋅ms or severe left atrial enlargement) was significantly higher in patients with ESUS compared to those with strokes caused by large artery atherosclerosis or small vessel disease [153]. Based on the above facts, several clinical studies [154, 155] have compared the effectiveness of non-vitamin K antagonist oral anticoagulants (NOACs) and aspirin in preventing secondary ischemic events in patients with initial ESUS. The multicenter, randomized, controlled, double-blind clinical trials NAVIGATE ESUS [154] and RE-SPECT ESUS [155] concluded that rivaroxaban or dabigatran were not better than aspirin in preventing recurrent stroke after an initial ESUS and was associated with a higher risk of bleeding events. However, patients after ESUS benefited from anticoagulation if the LA diameter was >4.6 cm based on the secondary analysis of NAVIGATE ESUS [18]. Furthermore, the ARCADIA trial [83] compared anticoagulation with antiplatelet therapy for secondary stroke prevention in patients with ESUS and evidence of ACM. In this study, ACM was assessed using a composite criterion (PTFV1 ≥5000 µV⋅ms, NT-proBNP ≥250 pg/mL, LA diameter index ≥3 cm/m^2^), and patients were randomly assigned to receive either aspirin or apixaban. The results, recently published in JAMA, showed that the rate of recurrent stroke during follow-up was 4.4% in both the apixaban and aspirin groups (HR 1.00; 95% CI 0.64–1.55) [83]. The results of the aforementioned studies indicate that in patients with clear evidence of ACM, the effectiveness of NOACs in preventing recurrent stroke is comparable to that of aspirin. Whether the combined use of aspirin and NOACs can further improve the prognosis of these patients without significantly increasing the risk of bleeding still requires further research to verify.

As previously mentioned, strokes can occur independently of AF. Therefore, accurately identifying individuals at high risk for stroke and initiating timely anticoagulant therapy is crucial. Notably, the CHA_2_DS_2_-VASc scoring system still remains effective in predicting the risk of ischemic stroke, even in the absence of a history of AF [156]. Furthermore, oral anticoagulants may provide benefits for ACM patients identified through abnormal imaging, ECG findings, and elevated inflammatory biomarkers, even those with low CHA_2_DS_2_-VASc scores [151]. For male patients with a CHA_2_DS_2_-VASc score of 1 and female patients with a score of 2, current AF clinical guidelines do not provide clear recommendations on anticoagulation therapy. Therefore, it is essential to consider whether the patient has concomitant ACM evidence when deciding whether to initiate anticoagulation. This consideration will help to more comprehensively weigh the benefits and risks of treatment.

### 4.3 Rhythm Control and “Reverse Remodeling” of Atrial Cardiomyopathy

Despite emerging evidence suggesting that stroke can occur in patients with AF even after sinus rhythm is restored [149], this does not imply that rhythm control is unimportant in the treatment of ACM. The EAST-AFNET 4 trial results indicated that early rhythm control therapy in early AF patients can reduce the risk of adverse cardiac outcomes including S/SE compared to usual treatment [157]. It is noteworthy that these patients received not only early rhythm control but also standardized anticoagulation therapy and comprehensive management of comorbid underlying cardiovascular diseases. These upstream treatments for ACM may be associated with better outcomes.

Furthermore, rhythm control itself may improve the substrate of ACM. In the early stages of AF, episodes typically originate from focal electrical activity mainly within muscle sleeves that extend into the pulmonary veins, usually manifesting as paroxysmal attacks. As the disease progresses, the atrial substrate undergoes continuous remodeling and deterioration, leading to a transition from paroxysmal to persistent AF, a process metaphorically referred to as “AF begets AF” [158, 159]. Therefore, maintaining sinus rhythm may be beneficial for atrial substrates. There is some evidence to suggest that the progression of ACM may be slowed or even reversed after effective catheter ablation. Sugumar *et al*. [160] published a subgroup analysis of the CAMERA-MRI study in the Journal of the American College of Cardiology: Clinical Electrophysiology, exploring the process of atrial electrical and structural remodeling after AF catheter ablation. They conducted detailed electroanatomical mapping of the right atrium using CARTO3 and contact force-sensing catheters on 15 patients who experienced a reduction of more than 90% in AF burden post-ablation. Over an average follow-up period of 23.4 ± 11.9 months, transthoracic echocardiography and CMR imaging revealed an improvement in LVEF and decreases in both right and left atrial areas. Additionally, electroanatomical (EA) mapping indicated a rise in right atrium bipolar voltage. Myocardial tissue voltage increased in all regions, with statistical differences observed in the posterior and septal segments [160]. Similarly, research by Pump *et al*. [161] suggested that catheter ablation is effective in patients with non-paroxysmal AF and severe LA enlargement, leading to reverse remodeling of LA and an improvement in left ventricular function.

However, the detrimental impact of additional damage caused by catheter ablation on atrial substrate remains unknown [162]. Extensive radiofrequency ablation generates additional scar tissue in the LA, and while this scar tissue may terminate current episodes of AF, its long-term effects on the atrial substrate are still uncertain. For patients with chronic systemic inflammatory or metabolic disorders, simple AF ablation cannot eradicate the pathophysiological mechanisms leading to ACM. In fact, the additional injury from the ablation procedure may further impair the atrial conduit, reservoir, and systolic functions. For patients already exhibiting significant signs of ACM before surgery, maintaining normal sinus rhythm after ablation is particularly challenging. Some physicians may opt for multiple ablations to eliminate AF, but the additional injury can further reduce LA ejection fraction and atrial compliance [163], exacerbate atrial fibrosis, and potentially lead to “stiff LA syndrome” [164]. As described above, the DECAAFII trail attempted to use LGE-CMRI to guide AF ablation, and 2 patients died post-ablation in the fibrosis-guided ablation group. In one case, the cause of death was likely due to excessive ablation of LA posterior wall [50]. Targeted ablation of fibrotic regions in LA may be a promising therapeutic strategy. Although the DECAAFII trail did not achieve statistically significant results, this study provides valuable insights for AF ablation strategies [50]. In the ERASE-AF study, PVI combined with individualized ablation of atrial low-voltage myocardium significantly improved ablation success rates and outcomes compared to PVI alone in patients with persistent AF [165]. Therefore, identifying which patients may derive long-term benefits from ablation surgery is especially crucial. This necessitates further research and validation through large-scale clinical trials.

## 5. Conclusions

Although several noninvasive tools exist to characterize ACM, clear diagnostic criteria are still lacking. Consequently, the concept of ACM remains largely in the realm of research and has not yet been translated into clinical practice guidelines. The pathogenesis of ACM is intricate and multifaceted, involving neural and humoral regulation, lipid accumulation, oxidative stress, and inflammatory responses. Given this complexity, interventional measures for ACM must be comprehensive. Future research should focus on developing multiple strategies to jointly intervene in the progression of ACM, particularly with targeted treatments that address the underlying mechanisms of the disease. Despite recent progress in ACM research, several key issues remain unresolved. These include establishing more precise and practical diagnostic criteria for ACM and determining whether anticoagulation therapy should be administered to individuals exhibiting characteristics of ACM but without AF. Additionally, further research is needed to identify which patients will benefit from AF ablation and whether patients with characteristics of ACM should continue long-term anticoagulation therapy after successful ablation. Addressing these issues is crucial for the clinical management of ACM and can help improve the overall prognosis of patients.
